# The role of macrophages-mediated communications among cell compositions of tumor microenvironment in cancer progression

**DOI:** 10.3389/fimmu.2023.1113312

**Published:** 2023-02-09

**Authors:** Mengyuan Li, Ping Jiang, Shuhua Wei, Junjie Wang, Chunxiao Li

**Affiliations:** Department of Radiation Oncology, Peking University Third Hospital, Beijing, China

**Keywords:** macrophages, tumor-associated macrophages, tumor microenvironment, crosstalk, cancer immunotherapy

## Abstract

Recent studies have revealed that tumor-associated macrophages are the most abundant stromal cells in the tumor microenvironment and play an important role in tumor initiation and progression. Furthermore, the proportion of macrophages in the tumor microenvironment is associated with the prognosis of patients with cancer. Tumor-associated macrophages can polarize into anti-tumorigenic phenotype (M1) and pro-tumorigenic phenotype (M2) by the stimulation of T-helper 1 and T-helper 2 cells respectively, and then exert opposite effects on tumor progression. Besides, there also is wide communication between tumor-associated macrophages and other immune compositions, such as cytotoxic T cells, regulatory T cells, cancer-associated fibroblasts, neutrophils and so on. Furthermore, the crosstalk between tumor-associated macrophages and other immune cells greatly influences tumor development and treatment outcomes. Notably, many functional molecules and signaling pathways have been found to participate in the interactions between tumor-associated macrophages and other immune cells and can be targeted to regulate tumor progression. Therefore, regulating these interactions and CAR-M therapy are considered to be novel immunotherapeutic pathways for the treatment of malignant tumors. In this review, we summarized the interactions between tumor-associated macrophages and other immune compositions in the tumor microenvironment and the underlying molecular mechanisms and analyzed the possibility to block or eradicate cancer by regulating tumor-associated macrophage-related tumor immune microenvironment.

## 1 Introduction

With the advancement of tumor immunology research, increasing cell subtypes have been identified in the tumor nest, and then the roles of the tumor microenvironment (TME) have attracted extensive attention (1). With the development of single-cell technologies, there is a new understanding of the importance of TME for tumor initiation and progression (2, 3). TME is a complex environment that is mainly composed of tumor cells and various immune cells, such as T cells, tumor-associated macrophages (TAMs), natural killer (NK) cells, neutrophils, dendritic cells (DCs), B lymphocytes and cancer-associated fibroblasts (CAFs) (4–9). Previous studies revealed that the interactions among various immune cells in the TME play an important role in tumor progression. The underlying mechanisms include gap junctions (10), receptors (11), release of small molecules (12), tunneling nanotubes (13), vesicles (14) and mechanical forces (15, 16). Furthermore, recent clinical trials have found that immunotherapy, which mainly relies on the activation of immune effector cells within TME by inhibiting immune checkpoints, has achieved a great success to improve the prognosis of patients with malignant tumors (17–19). Particularly, several immune checkpoint inhibitors to PD-1 and PD-L1 have been supplemented into the first-line treatment for some malignancies (20, 21).

TAMs are one of the most abundant cell types present in the TME of various cancers (22) and are tightly associated with other tumor infiltrated immune cells. Recent studies found that regulators of TAMs polarization and function can effectively modulate tumor progression (23). PD-1/PD-L1 and cytotoxic T lymphocyte antigen 4 (CTLA4) remain the most widely used targets of immune checkpoint inhibitors, which mainly regulating the immune functions of T cells (24–26), and increasing studies revealed that the number, activation status and polarization direction of TAMs are closely associated with the therapeutic efficacy (27–29). These findings suggest that TAMs have an irreplaceable effect in immunotherapy for cancers. In addition, chemotherapy coupled to macrophage-targeting strategies induces a more strong anti-tumor effect and achieves more tumor regressions in triple-negative breast cancer (29), pancreatic adenocarcinoma (30) and non-Hodgkin lymphoma (31). Meanwhile, macrophages inhibition combined with radiotherapy can also enhance anti-tumor effects (32, 33). Therefore, macrophages-based therapy may represent a novel approach for treating cancer. In this review, we summarized the characteristics of TAMs and the interactions between TAMs and other infiltrated immune cells, hoping to contribute to the understanding of TAMs and suggest effective ways related to TAMs-based modulation to block or eradicate cancers.

## 2 The characteristics of macrophage

### 2.1 The origin of macrophage

**Figure 1:**
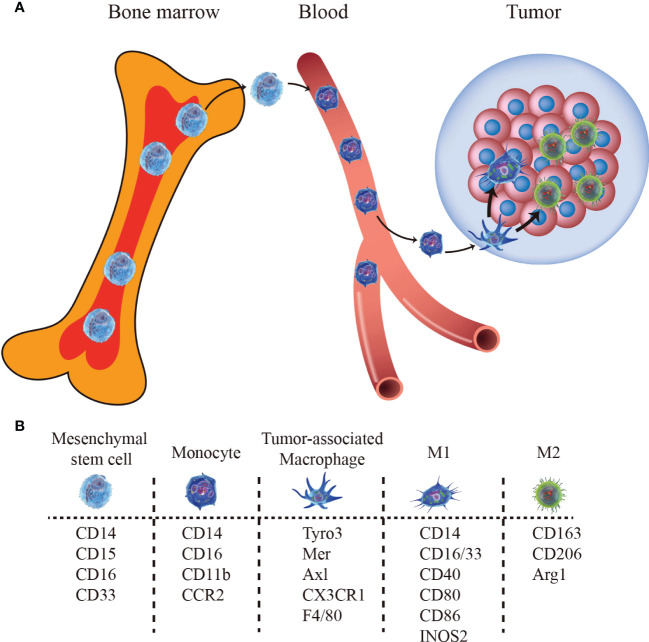
The bone marrow-derived macrophages pathway and biomarkers. **(A)** Monocytes egress from bone marrow and migrate to tumor sites, differentiating into TAMs. **(B)** Markers for mesenchymal stem cells, monocytes, TAM, M1-type TAM, and M2-type TAM.

Although the exact mechanism of macrophage formation remains controversial, two distinct lineages, bone marrow-derived macrophages, and tissue-resident macrophages have been widely recognized (34–38). Tissue-resident macrophages are embryonically derived and self-maintain locally (39), while bone marrow-derived macrophages are differentiated from monocytes originating from progenitors in the bone marrow which migrate from the bloodstream into tissues both in homeostasis and inflammation, following the stimulation of local growth factors, pro-inflammatory cytokines, and microbial products (**Figure 1**). Besides, these macrophage populations have a distinct temporal and spatial distribution in the TME (40). Tissue-resident macrophages spread to surrounding tumor cells early in the initial stages to promote epithelial-mesenchymal transition (EMT) and enhance the invasion, and they increase the number of regulatory T cells to promote the immune escape of tumor cells (40). Thus, the tissue-resident macrophages may be a novelly potential target for tumor therapy (38, 41). Phenotypically, different subtypes of macrophages can be identified by a set of overlapping and unique markers (**Figure 1**) and we have summarized them in **Table 1**.

**Table 1:** Phenotypic marker molecules of murine and human macrophage subsets.

Mouse				Human			
	Macrophage	M1	M2		Macrophage	M1	M2
CD45	+	+	+	CD45	+	+	+
CD11b	+	+	+	CD11b	+	+	+
CD11c	–	–	–	CD11c	+	+	–
CD68	+	–	–	CD68	+	–	–
F4/80	+	+	+	CD16	+/-	–	high
CD163	–	–	+	CD163	–	–	+
CD206	+	–	+	CD206	+	–	+
CD80	–	+	+	CD80	–	+	+
CD86	+	+	+	CD86	+	+	+
Ly6c	+	high	low				
MHC-II	+	+	+	HLA-DR	+	+	+
iNOS	–	+	–	iNOS	–	+	–
IL-1β	–	+	–	IL-1β	–	+	–
IL-8	–	+	–	IL-8	–	+	–
TNF-α	–	+	–	TNF-α	–	+	–
Arg-1	–	–	+	Arg-1	–	–	+
IL-10	–	–	+	IL-10	–	–	+
IL-12	–	+	–	IL-12	–	+	–
TGF-β	–	–	+	TGF-β	–	–	+
CD204	–	–	+	CD204	–	–	+

### 2.2 Macrophage polarization

Usually, macrophages are mainly polarized into two distinct subtypes to exert functions in regulating tumor progression. Lipopolysaccharide (LPS) together with pro-inflammatory cytokines such as Interferon-γ (IFN-γ) and tumor necrosis factor-α (TNF-α) assists the polarization of macrophages to the M1 phenotype (42, 43). Th1 cells are shown to be the major source of IFN-γ and TNF-α in the TME general inflammation. The alternatively activated macrophages (M2) are mainly activated by Th2 cytokines interleukin (IL)-4 and IL-13. The M1 macrophages secrete pro-inflammatory cytokines TNF-α, IFN-γ, IL-1β and IL-8 and exert pro-inflammatory and anti-tumor functions. While M2 macrophages mediate anti-inflammatory and tumorigenesis actions through producing anti-inflammatory factors transforming growth factor-beta (TGF-β), arginase 1 (Arg-1) and IL-10.

Moreover, recent findings revealed that M2 macrophages can be further divided into M2a, M2b, M2c and M2d subsets with distinct functions (**Table 2**). M2a-subset macrophages activated by IL-4 or IL-13 play an essential role in fibrosis, parasite killing and allergy. Both positive of CD206 and CD68 (CD206+/CD68+) is the character of M2a-subset macrophages. M2b-subset macrophages induced by immune complexes in combination with IL-1β or LPS play a vital role in immune response and are characterized by the expression of CD86 receptors (44, 45). M2c macrophages induced by IL-10, TGFβ or glucocorticoids exert a key role in anti-inflammatory and are characterized by the expression of CD163 receptors (46, 47). The M2d macrophages play an important role in tumor progression and are characterized by increased IL-10 and VEGF secretion and decreased expression of IL-12 and TNF-α, however, the specific mechanism underlying programming the M2d macrophages remains controversial (48, 49).

**Table 2:** M2-type macrophages subsets.

Subset	Stimuli	Markers	Functions
M2a	IL-4, IL-13	CD206, CD68	Anti-inflammatory
M2b	IL-1β, LPS	CD86	Immunoregulation, tumor progression
M2c	IL-10, TGFβ, glucocorticoids	CD163	Anti-inflammatory, angiogenesis, matrix remodeling, phagocytosis, wound healing
M2d	LPS, IL-6	IL-10, VEGF	Tumor progression, immunosuppressive, angiogenesis

## 3 Macrophages regulate tumor progression

**Figure 2:**
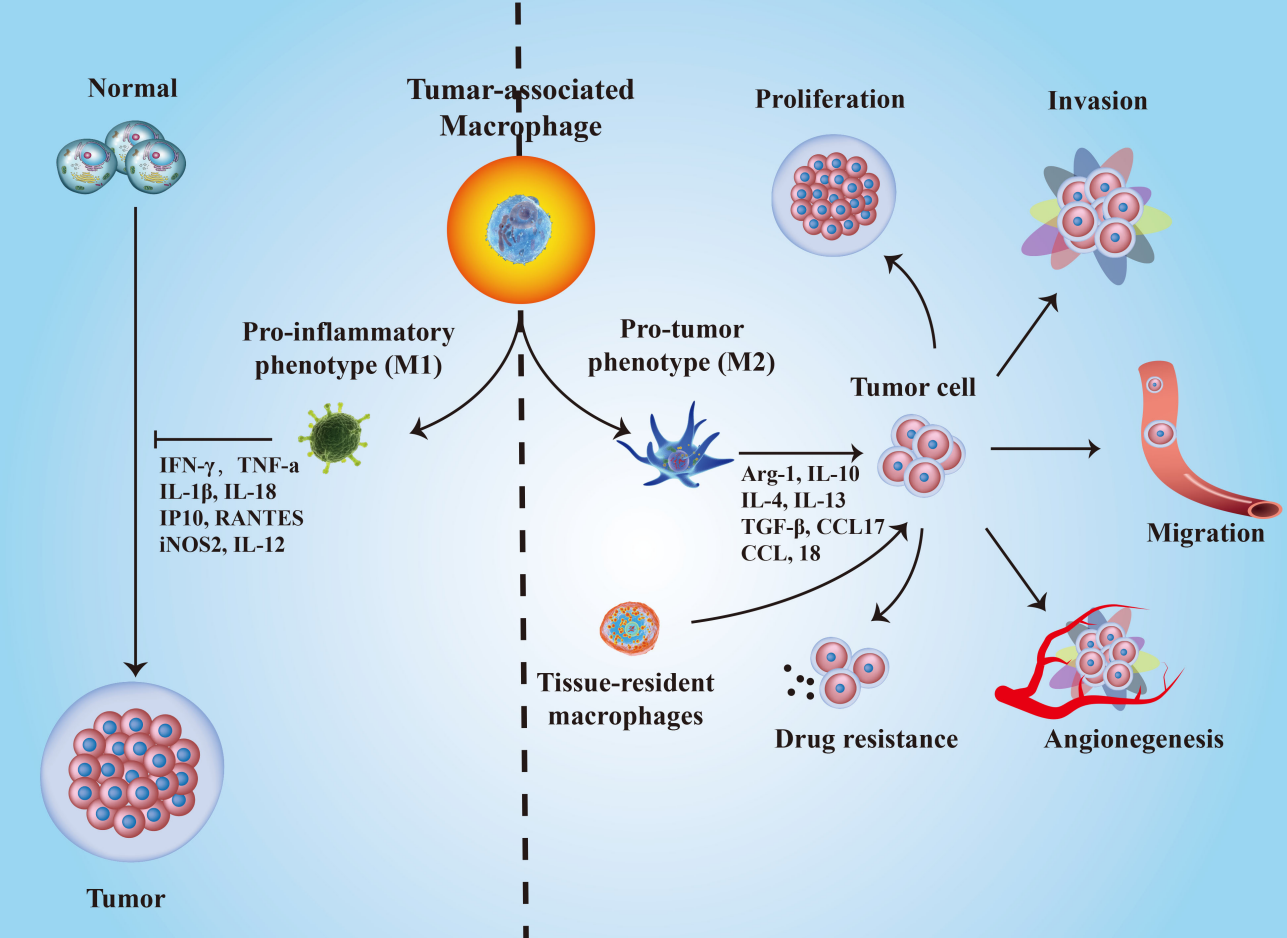
The roles of TAMs polarization in cancer progression. M1-type TAMs inhibit tumorigenesis by secreting IFN-γ, TNF-α, IL-18, IP10, IL-12, and iNOS2; while M2-type TAMs promote cancer development through several biological molecules, such as Arg-1, IL-10, IL-4, IL-13, TGF-β, CCL17, CCL-18, and so on.

Different directions of TAM polarization result in opposite functions in cancer progression. At the initial stages of tumor formation, macrophages mainly play a proinflammatory role and suppress tumor development, although the related evidence is still limited (50). As the tumor grows, macrophages in the TME are “educated” to a protumor phenotype by Th2 cells. Then, cytotoxic macrophages become tumor-supportive macrophages and promote tumor progression (51). A growing number of evidence suggested that TAMs exerted modulatory functions on tumorigenesis, progression, metastasis, angiogenesis and chemo-resistance (52) (**Figure 2**). For example, colony stimulating factor-1 (CSF-1) promotes malignant transformation in mammary cancer by recruiting macrophages (53). TAMs can also facilitate tumor cell intravasation and extravasation by secreting epidermal growth factor (EGF) (54) and vascular endothelial growth factor (VEGF) (55). EGF secreted by TAMs promotes tumor cell intravasation into blood vessels, while VEGF triggers endothelial cell barrier disruption by destroying adherens junctions. In addition, TAMs modulate tumor metastasis by regulating the EMT process through STAT/miR-506-3p/FoxQ1 signaling and TAT/miR-506-3p/FoxQ1 pathway and promote extracellular matrix degradation *via* secreting matrix metalloproteinases and C-C motif chemokine ligand 18 (CCL18) (56–60). Besides, M1-type TAMs can exert a direct killing effect on tumor cells once activated by IFN-γ or mediate adaptive immunity by recruiting and activating CD8^+^ T cells and NK cells after presenting tumor antigens and producing chemokines and cytokines (50). Majority of the TAM’s in the TME tends to be M2 but not M1 thereby shifting the antitumor microenvironment to an immunosuppressive milieu (61). However, several studies reached opposite conclusions. For example, M2 macrophages may have a partial limiting effect on colorectal cancer metastasis (62), whereas M1 macrophages promote tumor progression (63, 64). Therefore, the characteristics of TAMs contribute to better understanding the cancer states and exploring new ways to block or eradicate cancers.

## 4 Interactions between TAMs and other cell components in the TME

**Figure 3:**
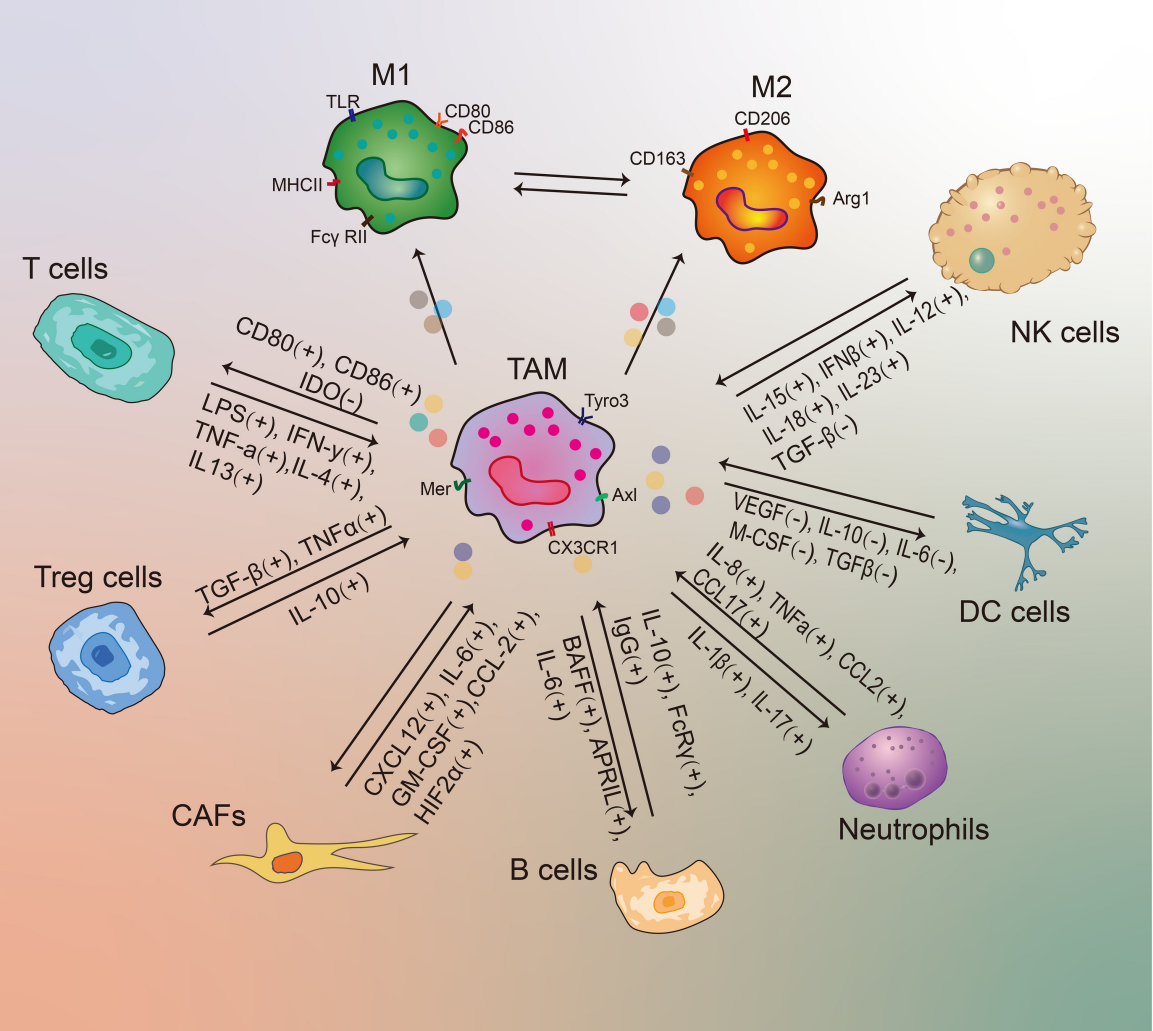
The molecular interactions between TAMs and other immune cells in the TME. A “+” or “-” sign shows a stimulatory or inhibitory interaction.

Recently, substantial studies revealed that TAMs play a pivotal role in regulating tumor development through interacting with various immune cells in TME (50). For example, M2-phenotype TAMs gradually becomes the major TAM under the stimulation of Th2 cells, and then the antitumor functions of TAMs are diminished (61). In addition, M1-type TAMs can exert a killing effect on tumor cells once activated by IFN-γ (50). TAMs can also express T-cell immune checkpoint ligands, such as PD-L1, CD80 and CD86, to inhibit T-cell functions (50, 65). Therefore, it is important to better understand the interactions between TAMs and other immune cells in the TME. The detailed interactions between other TME contents and TAMs were further described as follows (**Figure 3**).

### 4.1 TAMs and T cells

Both TAMs and T cells can polarize into different subtypes with different functions under diverse signals’ stimulation. On the one hand, the polarization of TAMs is mainly regulated by T cells, especially Th1 and Th2 cells. On another hand, during antigen presentation, macrophages can also simultaneously activate multiple T cells (66). T cells perceive the stimulatory signals through interacting with macrophages and are eventually activated. Therefore, macrophages play an important role in activating T cells. Different macrophages subtypes may have different functional effects on T cells. For example, the M1 macrophages can activate T cells *via* upregulating B7 receptors, such as CD80 (B7-1) and CD86 (B7-2), while M2 macrophages are not able to express costimulatory molecules of the B7 family but exert disruption function through binding with T cells (67). FOLR2^+^ macrophages, which are located in the perivascular spaces in the tumor tissue, can effectively activate the CD8^+^ T cells in the tumor nest, and then improve the prognosis of breast cancer patients (68). In addition, macrophages also inhibit T cell proliferation *in vitro* through indoleamine 2,3-dioxygenase (IDO)-induced tryptophan degradation (69). The cavity-resident macrophages with high levels of Tim-4 can weaken the efficacy of anti-PD1 therapy in lung cancer by reducing PD-1 expression levels in CD8^+^ T cells (70). Moreover, the levels of TAMs-secreted TGF-β are significantly elevated in malignant pleural effusion, which plays an important role in destroying T cell function and promoting cancer progression in lung cancer patients (12).

Recent studies have confirmed that TAMs could produce large amounts of extracellular vesicles to influence the biological function of tumor cells and T cells in TME through extracellular vesicles fusion or cell-cell membrane contact (71). Notably, though TAMs exert immunosuppressive in various cancers, TAMs-derived extracellular vesicles can promote T cells proliferation and activation and exhibit M1 macrophages characteristics in colorectal tumor (71). However, M2 macrophages-derived extracellular vesicles induce CD8^+^ T cells exhaustion and promote tumor progression in hepatocellular carcinoma (72). Wang et al. introduced the nucleus of tumor cells into M1 macrophages to create chimeric exosomes, the chimeric exosomes can enter lymph nodes and induce T cells activation through direct exosome contact or antigen presenting cells induced immunostimulatory manner (14).

In addition to producing soluble secreted factors and extracellular vesicles, macrophages also modulate functions of T cells through direct interaction. A new research found that there were unique, antigen-specific synaptic interactions between TAMs and CD8^+^ T cells through using lattice light sheet microscopy (73). These interactions were unable to activate T cells, but result in exhaustion of T cells, which is significantly enhanced under hypoxic conditions (73). Therefore, seeking effective ways to target both macrophages and T cells may be a promising approach to improving the efficacy of immunotherapy.

### 4.2 TAMs and Tregs

Treg cells are a subset of CD4^+^ T cells which play a key role in tumor-associated immunosuppression (74). These cells are defined by the characteristic of the expression of transcription factor Foxp3 and IL-12 receptor α-chain (CD25). In inflammation resolution, Tregs stimulate macrophages’ efferocytosis *via* the production of IL-10 and induce apoptotic cell internalization (75). They can efficiently promote macrophages’ polarization into the M2 phenotype and downregulate the immune response (76, 77). Meanwhile, macrophages can maintain Tregs proliferation to suppress type 2 inflammatory responses (78). Furthermore, Kraaij et al. found that Tregs induced by macrophages are regulated *via* macrophages-derived reactive oxygen species (ROS) (79).

Many studies have established that Tregs promote tumor progression, such as hepatocellular carcinoma (80), breast cancer (81) and esophageal squamous cell carcinoma (82). Studies have also revealed a significant association between macrophages and Tregs in tumor progression (83). For example, TAMs induce the conversion of CD4^+^ T cells into Tregs through secreting TGF-β and promoting PD-1 expression on CD4^+^ T cells, resulting in Tregs infiltration in tumors (84). M2 macrophages can activate the TGF-β/Smad signaling pathway by expressing TGF-β, then induce Tregs generation and promote colorectal cancer development (85). In epithelial ovarian cancer, TAMs can upregulate Treg/Th17 ratios and promote tumor progression through releasing exosomes that contain miR-29a-3p and miR-21-5p targeting STAT3 to T cells (86). In addition, Liu et al. found that Tregs facilitate the M2-polarization of macrophages through inhibiting CD8^+^ T cells expression of IFN-γ and activating M2 macrophages sterol regulatory element-binding protein 1 mediated fatty acid synthesis (87). Thus, targeting Tregs and TAMs interaction may be an effective anti-tumor approach. In addition, radiotherapy is considered one of the most important treatment modalities in clinic. It is well recognized that radiotherapy induces inflammatory cells recruitment into TME, as well as immunosuppressive cells (88). Mondini and colleagues confirmed that radiotherapy can promote the secretion of CCL2 by tumor cells and induce the accumulation of CCR2^+^ Tregs and CCR2-dependent macrophages which can produce TNF-α, then TNF-α induces Tregs activation and decreases the efficacy of radiotherapy (89). Therefore, CCL2/CCR2 inhibitors in combination with radiotherapy may be an efficient approach for improving the therapeutic effects of radiotherapy in tumor treatment.

In addition to potential therapeutic targets, macrophages and Tregs infiltration can also be used as a prognostic biomarker for tumors. For example, the high level expression of Tregs indicates a better prognosis in early-stage gastric cancer patients, while the opposite results have been found in late-stage patients (90). Meanwhile, M2 macrophages predict a worse prognosis in general, however, high infiltration of M2 macrophages suggests a good prognosis in signet ring cell carcinoma and mucinous adenocarcinoma. The combination of both indicators can improve the prediction accuracy of cancers. In addition, single-cell RNA sequencing (scRNA-seq) revealed that M2 macrophages and Tregs infiltration are adverse prognostic factors for prostate cancer patients (91), colorectal cancer (92) and hepatocellular carcinoma patients (93). Thus, the specific prognostic value of TAMs and Tregs should be investigated in different cancers.

### 4.3 TAMs and CAFs

Activated fibroblasts in tumors are defined as CAFs (94). Though the origin of CAFs remains controversial, some researchers proposed that CAFs can derive from tissue-resident fibroblast, bone-marrow-derived mesenchymal stem cells (95), pancreatic or hepatic stellate cells (96, 97), adipocytes (98) and endothelial cells (99). CAFs exhibit a wide range of phenotypic and functional heterogeneity, and there is no clear biological marker to identify CAFs at present (100, 101). The functions of CAFs in tumor progression have been widely studied. For example, CAFs have been demonstrated to promote tumorigenesis and metastasis in breast cancer (102), lung cancer (103) and colorectal carcinoma (104). Moreover, CAFs have also been found to exert anti-tumor effects in pancreatic cancer (105).

As TAMs and CAFs are both major components in TME and TAMs infiltration increases in the regions where CAFs are enriched, there might exist a tight correlation between them (106, 107). Studies have revealed that CAFs can regulate TAMs infiltration in TME and induce TAMs to polarize into a pro-tumorigenic phenotype (107). For example, Zhang et al. found that CAFs can induce TAMs infiltration and promote M2 macrophage polarization, which leads to loss of NK cells function and contributes to an immune suppressive environment in colorectal cancer (108). Furthermore, a similar effect of CAFs on TAMs was found in hepatocellular carcinoma through secreting CXCL12 (109). Similarly, CAFs can also stimulate TAMs through other cytokines, such as IL-6, GM-CSF (110) and CCL2 (111). In addition to cytokines, CAFs can also regulate M2 macrophage polarization through expressing hypoxia inducible factor 2α (HIF2α) and promote pancreatic cancer progression (112). Meanwhile, CAFs not only promote TAMs infiltration and polarization but also enhance TAMs expression of PD-1, which leads to decreased phagocytosis and enhanced immunosuppressive functions (113, 114).

TAMs also regulate CAFs functions and activation as well. For example, Tang et al. revealed that TAMs promote CAFs generation *via* Smads-mediated macrophage-myofibroblast transition (115). Meanwhile, M2 macrophages can enhance CAFs activation by regulating the mesenchymal-mesenchymal transition of fibroblasts (115) and secreting TGF-β (116). Then, activated CAFs further enhance TAMs recruitment and activity, resulting in an immunosuppressive environment. Collectively, the interaction between TAMs and CAFs generates a cancer-promoting phenotype. However, the exact mechanism of CAFs and TAMs interaction remains undefined, further investigation is required for therapeutic exploitation.

### 4.4 TAMs and B cells

Recently, several studies have revealed that high levels of B cells in the tumor nest indicate a persistent immune activation response and predict a good efficacy of immunotherapy for patients with cancer (117). Furthermore, TAMs and B cells have a close association. For example, subcapsular-sinus macrophages play an important role in accumulating various larger antigens through the expression of sulphated glycoproteins which can preserve the integrity of antigens and then present antigens to the neighboring follicular B cells (118–120). Meanwhile, macrophages can regulate the transportation and retention of B cells in the splenic marginal zone (121).

Besides, tissue-resident macrophages are not only derived from monocytes but can also differentiate from early pro-B cell/fraction B within the bone marrow, these macrophages’ precursors enter into the systemic circulation and acquire the same transcriptome identical as embryonically derived macrophages (122). These macrophages precursors also gain CD115, F4/80, and CD16/32 after entering inflammation sites, which are very similar to blood monocyte-derived macrophages (122). Thus, pre/pro-B cells may be an additional source of macrophages. It is worth noting that B1 cells can migrate into the inflammatory milieu and differentiate into a macrophage-like cell type *in vitro* (123). In turn, macrophages can also regulate B cell proliferation *via* secreting B cells-activating factor (BAFF) and a proliferation-inducing ligand (APRIL) (124). Macrophages have also been verified to support the later B1 cells development *via* expressing IL-6 (125). Meanwhile, B cells can also regulate the polarization of macrophages. For example, B cells induce peritoneal macrophages to polarize into an M2-like phenotype through secreting IL-10 and this phenomenon is also observed in tumors where B cells reprogram TAMs into the M2 macrophages (126). In addition, Andreu et al. found that B cells promote the M2 macrophage infiltration and induce the proangiogenic and protumorigenic effects of macrophages through activating Fc-gamma (FcRγ) in squamous carcinoma (127). Chemokines receptors trigger B cells migration into lymphoid follicles, such as chemokine (C-X-C motif) receptor 4 (CXCR4) and CXCR5 (128). Liu and colleagues demonstrated that CXCR3^+^ B cells infiltrate predominantly in hepatocellular carcinoma invading edge and are associated with tumor recurrence, furthermore, CXCR3^+^ B cells induce TAMs repolarization into M2 macrophages through an IgG-dependent manner and promote hepatocellular carcinoma progression (129).

Collectively, current studies have revealed that TAMs and B cells have associations in their origins and influence each other. However, the detailed mechanisms remain unclear and still require further clarification.

### 4.5 TAMs and neutrophils

Neutrophils are phagocytic cells that are an important part of the innate immune system and play a pivotal role in the first–line of defense (130). Like TAMs, tumor–associated neutrophils (TANs) can also polarize into anti–tumor (N1) phenotype and pro–tumor (N2) phenotype according to different cytokines stimuli (131). Considering that neutrophils can recruit macrophages via secreting IL–8 and TNF–α in an inflammatory environment and macrophages can in turn regulate neutrophils function, the TAMs and TANs may have close interrelationships during tumor progression (132). For example, TAMs induce IL–17 production through releasing IL–1β, the IL–17 can enhance neutrophils recruitment and promote tumor metastasis in breast cancer (133, 134). Similarly, TANs promote TAMs and T–regulatory cell recruitment in hepatocellular carcinoma via secreting CCL2 and CCL17, leading to tumor growth and drug resistance (135). Meanwhile, both TAMs and TANs can produce matrix metalloproteinase–9, which releases angiogenic factors and VEGF to promote angiogenic (136).

Besides, growing evidence has found that the neutrophils-to-lymphocytes ratio is a prognostic biomarker in patients with pancreatic tumors (137), colorectal cancers (138) and hepatocellular carcinoma (139), and a higher ratio predicts a poor prognosis. The accumulation of TAMs in TME can elevate the neutrophils-to-lymphocytes ratio and confers a poorer prognosis for patients (137). Furthermore, Huang et al. found that a combination of CD163^+^ TAMs and CD66b^+^ TANs is an important prognostic marker for gastric cancer patients (140).

### 4.6 TAMs and DCs

Macrophages and DCs are forefront cells of innate immunity, they are capable of sensing and immediately against invading pathogens (141). Though macrophages and DCs are different cell types and originate from different lineages, they express several same markers and exhibit some similar functions (142). For example, macrophages and DCs are both found in peripheral tissues and accumulate in the areas of pathogen entry (143). Besides, macrophages and DCs can exert a synergetic effect on connecting innate and adaptive immunity through recognizing and presenting the foreign antigens to T cells (144–146). The phagosomal degradation of DCs is lower than macrophages, which retain the antigenic peptides and initiates adaptive immune responses (143).

ScRNA-seq analyses found that macrophages and DCs play a key role in mediating cellular cross-talk in the TME and regulate tumor immunity (147). The potent anti-tumor immune response needs antigen presentation by macrophages and DCs. Immature DCs can get matured and migrate from the periphery to the lymph node and activate T cells when they recognize pathogen-associated molecular patterns (PAMPs) and damage-associated molecular patterns (DAMPs) (148). Whereas, the maturation and function of DCs can be inhibited by several factors secreted by tumor cells and TAMs, such as VEGF, IL-10, IL-6, M-CSF and TGFβ (149–153). Furthermore, a study revealed that the immature or defective DCs results in T cells’ unresponsiveness and immunosuppression in TME (154). DAMPs have been referred to endogenous molecules and fragments from damaged cells and tissues, which were also be recognized as danger signals (155, 156). The adenosine triphosphate (ATP), which is also an important component of TME, is likely to be the prototypical and most widely diffused DAMPs (157). Studies have confirmed that ATP can not only promote DCs migration into lymph nodes and activate T cells, but also regulate TAMs physiology (158–160). Regarding the proteins involved in this ATP-related signaling, there are Connexins and Pannexins channels, which allow contact dependent or independent communication (160–162). Interestingly, Pannexins are differentially expressed during macrophage polarization, which makes them valuable target for therapy (163). In advanced osteosarcoma, Zhou et al. found that monocytes and macrophages make up the majority of total myeloid cells at 70-80%, while DCs only account for less than 5% by scRNA-seq analysis (164). It is still uncovered whether the decreasing proportion of DCs is associated with poor prognosis in tumors. Currently, there are limited studies exploring the interaction of DCs and macrophages on the effects of tumors, targeting the cross-talk between DCs and macrophages may be an effective anti-tumor strategy.

### 4.7 TAMs and NK cells

NK cells are also an important component of innate immunity which play a pivotal role in the defense against infections and cancer (165). NK cells can also promote TAMs to repolarize into the M1-type macrophages (166). The cross-talk between macrophages and NK cells have been verified as an important part of inflammatory and anti-tumor reactions. Macrophages promote NK cell activation mainly through secreting cytokines, such as IL-15, IFNβ (167), IL-12, IL-18 (168) and IL-23 (169). Once activated, NK cells produce large amounts of IFN-γ to exert cytotoxic effects. Besides, M1 macrophages increase NK cells number and induce NK cell activation to express TNF-related apoptosis-inducing ligand which can promote hepatic stellate cell apoptosis in the fibrotic liver (170). In contrast, TAMs can also inhibit NK cell function through expressing TGF-β (171).

Notably, NK cells exhibit distinct functions when interacting with different phenotypes of TAMs. For example, activated NK cells can kill M0- and M2-TAMs, while the M1-TAMs are more resistant to lysis than M0- and M2-TAMs due to their high levels of HLA class I molecules (172). Besides, after stimulation with LPS, M0- and M2-TAMs induce the activation of resting NK cells and promote the expression of CD69, CD25 and CCR7.

### 4.8 TAMs and NKT cells

Natural killer T (NKT) cells are a unique lymphocyte population which can recognize lipid antigens presented by the MHC class I-like molecular CD1d (173). Upon activated by CD1d, NKT cells initiated an essential role in autoimmunity, infection and tumor immunity through secreting a lot of cytokines, including TNF-α, IFN-γ, IL-4, IL-6 and IL-17 (174–177). Furthermore, the activated NKT cells also increase the proportion of M1-type macrophages and reduce M2 macrophages in the TME to exert an antitumor effect (173). In addition, recent study found that TAMs can promote tumor growth through producing IL-6, but accounting for majority of CD1d-expressing cells (178). Further mechanismic research revealed that CD1d-activated NKT cells can recognize TAMs specifically and kill TAMs to suppress tumor growth (178). Therefore, NKT-based therapies that can against both tumor cells and TAMs will be an effective antitumor treatment.

## 5 Cancer cell therapy by targeting TAMs-based communications among TME

Since the first chimeric antigen receptor (CAR)-T cell therapy (Kymriah) was approved by FDA, the cell therapy field is still expanding and evolving (179). Although CAR-T therapy has achieved remarkable success in hematological malignancies, the efficacy of CAR-T treatment of solid tumors is limited (180, 181). Therefore, it is urgent to find more effective cellular immunotherapeutic strategies. Currently, the unique characteristics of macrophages make it a proper candidates for the treatment of solid tumors (182). CAR macrophages (CAR-M) demonstrated antigen-specific phagocytosis and increase antigen-presentation ability. Meanwhile, CAR-M can also reprogram M2 macrophages to M1 and stimulate the expression of pro-inflammatory cytokines and chemokines to induce a pro-inflammatory microenvironment and enhance T cell-mediated antitumor activity (182, 183). Nevertheless, clinical trials and results about CAR-M have been highly limited, there is still a long way to go for CAR-M therapy (184). Given that the crucial role of TAMs in cancer progression and response to treatment, TAMs-based cell therapies have been well studied and the combination therapeutic strategies in clinical trials are included in **Table 3**.

**Table 3:** The combination therapy targeting on TAMs in clinical trials.

Targets	Drugs	Clinical Phase	Conditions	Combinations in trials	Sponsor	Gov identifier
CSF1	MCS110	1	Breast Cancer	Doxorubicin	Washington University School of Medicine	NCT03285607
				Paclitaxel		
				Doxorubicin		
		1/2	Triple Negative Breast Cancer	PDR001	Novartis Pharmaceuticals	NCT02807844
			Pancreatic Carcinoma			
			Melanoma			
			Endometrial Carcinoma			
		1/2	Melanoma	Dabrafenib	Dana-Farber Cancer Institute	NCT03455764
				Trametinib		
	PD-0360324	2	Recurrent Fallopian Tube Carcinoma	Cyclophosphamide	M.D. Anderson Cancer Center	NCT02948101
			Recurrent Ovarian Carcinoma			
			Recurrent Primary Peritoneal Carcinoma			
CSF1R	LY3022855	1	Solid Tumor	Durvalumab or Tremelimumab	Eli Lilly and Company	NCT02718911
			Neoplasms	NR	Eli Lilly and Company	NCT01346358
		1	Pancreatic Cancer	Pembrolizumab	Sidney Kimmel Comprehensive Cancer Center at Johns Hopkins	NCT03153410
		1/2	Melanoma	Vemurafenib	Dana-Farber Cancer Institute	NCT03101254
				Cobimetinib		
	PLX3397	1/2	Melanoma	Pembrolizumab	Daiichi Sankyo, Inc.	NCT02452424
			Non-small Cell Lung Cancer			
			Squamous Cell Carcinoma of the Head and Neck			
			Gastrointestinal Stromal Tumor (GIST)			
			Ovarian Cancer			
	Cabiralizumab	2	Pancreatic Cancer Stage IV	Gemcitabine	Hitendra Patel	NCT03697564
				Nivolumab		
		1	Advanced Melanoma	APX005M	Yale University	NCT03502330
			Advanced Melanoma	Nivolumab		
			Renal Cell Carcinoma			
		2	Head and Neck Squamous Cell Carcinoma	Nivolumab	Sidney Kimmel Comprehensive Cancer Center at Johns Hopkins	NCT04848116
		2	Resectable Biliary Tract Cancer	Nivolumab	Sidney Kimmel Comprehensive Cancer Center at Johns Hopkins	NCT03768531
	Edicotinib	2	Recurrent Acute Myeloid Leukemia			
	DCC3014	1	Sarcoma	Avelumab	Memorial Sloan Kettering Cancer Center	NCT04242238
			Advanced Sarcoma			
			High Grade Sarcoma			
			Leiomyosarcoma			
			Leiomyosarcoma			
			Leiomyosarcoma			
			Dedifferentiated Liposarcoma			
	ARRY-382	2	Advanced Solid Tumors	Pembrolizumab	Pfizer	NCT02880371
	SNDX-6352	2	Unresectable Intrahepatic Cholangiocarcinoma	Durvalumab	Sidney Kimmel Comprehensive Cancer Center at Johns Hopkins	NCT04301778
CSF1R-TKI	pexidartinib	1	Clorectal Cancer	Durvalumab	Centre Leon Berard	NCT02777710
			Pancreatic Cancer			
			Metastatic Cancer			
			Advanced Cancer			
CCR2	PF-04136309	2	Metastatic Pancreatic Ductal Adenocarcinoma	Nab-paclitaxel	Pfizer	NCT02732938
				Gemcitabine		
CCR2/CCR5	BMS-813160	2	Non-small Cell Lung Cancer	Nivolumab	Icahn School of Medicine at Mount Sinai	NCT04123379
			Hepatocellular Carcinoma	BMS-986253		
		1/2	Locally Advanced Pancreatic Ductal Adenocarcinoma (PDAC)	Stereotactic Body Radiation	Sidney Kimmel Comprehensive Cancer Center at Johns Hopkins	NCT03767582
			Pancreatic Ductal Adenocarcinoma	Nivolumab		
				GVAX		
		1/2	Pancreatic Ductal Adenocarcinoma	Nivolumab	Washington University School of Medicine	NCT03496662
				Gemcitabine		
				Nab-paclitaxel		
CCR5	Maraviroc	1	Metastatic Colorectal Cancer	Pembrolizumab	University Hospital Heidelberg	NCT03274804
CD40	APX005M	1	Advanced Melanoma	Cabiralizumab		
			Advanced Melanoma	Cabiralizumab		
			Renal Cell Carcinoma			
		1/2	Non Small Cell Lung Cancer Metastatic	Nivolumab	Apexigen, Inc.	NCT03123783
			Metastatic Melanoma			
			Neoplasm of Lung			
			Melanoma			
		2	Soft Tissue Sarcoma	Doxorubicin	Columbia University	NCT03719430
		1/2	Melanoma	Pembrolizumab	M.D. Anderson Cancer Center	NCT02706353
	CDX-1140	1/2	Melanoma	Poly-ICLC	Craig L Slingluff, Jr	NCT04364230
		1/2	Non Small Cell Lung Cancer	SBRT	Albert Einstein College of Medicine	NCT04491084
			Lung Cancer			
	SGN-40	1	Multiple Myeloma	lenalidomide	Seagen Inc.	NCT00525447
				dexamethasone		
		1	Multiple Myeloma	bortezomib	Genentech, Inc.	NCT00664898
CD47	AK117	1/2	Acute Myeloid Leukemia	Azacitidine	Akeso	NCT04980885
		1/2	Myelodysplastic Syndrome	Azacitidine	Akeso	NCT04900350
	ALX148	2	Microsatellite Stable Metastatic Colorectal Cancer	Cetuximab	Criterium, Inc.	NCT05167409
				Pembrolizumab		
		2/3	Gastric Cancer	Trastuzumab	ALX Oncology Inc.	NCT05002127
			Gastroesophageal Junction Adenocarcinoma	Ramucirumab		
			Gastric Adenocarcinoma	Paclitaxel		
		2	Head and Neck Cancer	Pembrolizumab	ALX Oncology Inc.	NCT04675333
			Head and Neck Squamous Cell Carcinoma	Cisplatin/Carboplatin; 5FU		
		2	Head and Neck Cancer	Pembrolizumab	ALX Oncology Inc.	NCT04675294
			Head and Neck Squamous Cell Carcinoma			
		1/2	Aggressive B-Cell Non-Hodgkin Lymphoma	Lenalidomide	M.D. Anderson Cancer Center	NCT05025800
			Ann Arbor Stage III Grade 2 Follicular Lymphoma			
			Ann Arbor Stage III Grade 3 Follicular Lymphoma			
	TTI-622	1/2	Ovarian Cancer	Pegylated Liposomal Doxorubicin	Trillium Therapeutics Inc.	NCT05261490
			Ovarian Neoplasms			
			Ovarian Carcinoma			
			Fallopian Tube Cancer			
			Fallopian Tube Cancer			
			Primary Peritoneal Carcinoma			
		1/2	Leiomyosarcoma	Doxorubicin	Trillium Therapeutics Inc.	NCT04996004
			Myelodysplastic Syndromes			
	TG-1801	1	Marginal Zone Lymphoma	Ublituximab	TG Therapeutics, Inc.	NCT04806035
			Follicular Lymphoma			
			Aggressive Lymphoma			
	Magrolimab	2	Hodgkin Lymphoma	Pembrolizumab	Stanford University	NCT04788043
			Classic Hodgkin Lymphoma			
			Relapsed Classical Hodgkin Lymphoma			
			Refractory Classic Hodgkin Lymphoma			
	SL-172154	1	Cutaneous Squamous Cell Carcinoma	NR	Shattuck Labs, Inc.	NCT04502888
			Squamous Cell Carcinoma of Head and Neck			
PI3K	Alpelisib	2	Breast Cancer, PI3K, Alpelisib	Chemotherapy	UNICANCER	NCT03386162
	BKM120	1	Recurrent Non-small Cell Lung Cancer	pemetrexed disodium	City of Hope Medical Center	NCT01723800
			Stage IV Non-small Cell Lung Cancer	carboplatin		
		1/2	Breast Cancer	Lapatinib	Institut Paoli-Calmettes	NCT01589861
		1	Extensive Stage Small Cell Lung Cancer	cisplatin	University of California, Davis	NCT02194049
			Unspecified Adult Solid Tumor, Protocol Specific	etoposide		
		1	Unspecified Adult Solid Tumor, Protocol Specific	docetaxel	Roswell Park Cancer Institute	NCT01540253
		1/2	Metastatic Squamous Neck Cancer With Occult Primary Squamous Cell Carcinoma	cetuximab	University of Chicago	NCT01816984
			Recurrent Metastatic Squamous Neck Cancer With Occult Primary			
			Recurrent Salivary Gland Cancer			
		2	Advanced Prostate Cancer	Cabazitaxel	SCRI Development Innovations, LLC	NCT02035124
	BYL719	1	Advanced Gastric Cancer	AUY922	Novartis Pharmaceuticals	NCT01613950
		1	Estrogen Receptor-positive Breast Cancer	letrozole	Vanderbilt-Ingram Cancer Center	NCT01791478
			HER2-negative Breast Cancer			
			Invasive Ductal Breast Carcinoma			
	Copanlisib	2	Endometrial Cancer	Fulvestrant	M.D. Anderson Cancer Center	NCT05082025
			Ovarian Cancer			
		1/2	Colon Cancer	Nivolumab	Sidney Kimmel Comprehensive Cancer Center at Johns Hopkins	NCT03711058
	PF-05212384	1	Advanced Cancer	PD-0325901	Pfizer	NCT01347866
				Irinotecan		
	Duvelisib	1	Chronic Lymphocytic Leukemia	Venetoclax	AbbVie	NCT02640833
			Small Lymphocytic Lymphoma			
			Non-Hodgkin Lymphoma			
TLR						
TLR7	Imiquimod	1/2	Breast Cancer	Cyclophosphamide	NYU Langone Health	NCT01421017
			Metastatic Breast Cancer			
			Recurrent Breast Cancer			
		1	Melanoma (Skin)	indocyanine green solution	University of Oklahoma	NCT00453050
			Metastatic Cancer			
	RO7119929	1	Carcinoma, Hepatocellular	Tocilizumab	Hoffmann-La Roche	NCT04338685
			Biliary Tract Cancer			
			Secondary Liver Cancer			
			Liver Metastases			
	SHR2150	1/2	Solid Tumor	Anti-Cancer Agent	Chinese PLA General Hospital	NCT04588324
						
	BNT411	1/2	Solid Tumor	Atezolizumab	BioNTech SE	NCT04101357
			Extensive-stage Small Cell Lung Cancer	Carboplatin		
TLR9	MGN1703	1	Advanced Cancers	Ipilimumab	M.D. Anderson Cancer Center	NCT02668770
			Melanoma			
	Tilsotolimod	1	Advanced Cancer	Ipilimumab	Gustave Roussy, Cancer Campus, Grand Paris	NCT04270864
				Nivolumab		
	SD-101	1	Advanced Malignant Solid Neoplasm	BMS 986178	Ronald Levy	NCT03831295
			Extracranial Solid Neoplasm			
			Metastatic Malignant Solid Neoplasm			
		1	Metastatic Pancreatic Adenocarcinoma	Nivolumab	University of California, Davis	NCT04050085
			Refractory Pancreatic Adenocarcinoma			
			Pancreatic Cancer			
		1	Metastatic Uveal Melanoma in the Liver	Nivolumab	TriSalus Life Sciences, Inc.	NCT04935229
				Ipilimumab		
	IMO 2055	1	Colorectal Cancer Metastasis	Cetuximab	EMD Serono	NCT00719199
				FOLFIRI		
	CMP-001	2	Melanoma	Nivolumab	Diwakar Davar	NCT03618641
			Lymph Node Cancer			
		2	Melanoma	Nivolumab	Diwakar Davar	NCT04401995
			Relapsed Acute Myelogenous Leukemia			
	IMO-2125	3	Metastatic Melanoma	Ipilimumab	Idera Pharmaceuticals, Inc.	NCT03445533
TLR4	GLA-SE	1	Colorectal Cancer Metastasis	FOLFOX regimen	Gustave Roussy, Cancer Campus, Grand Paris	NCT03982121
				Nivolumab		
				Ipilimumab		
	GSK1795091	1	Cancer	Placebo	GlaxoSmithKline	NCT02798978
			Neoplasms			
TLR8	VTX-2337	1	Colorectal Adenocarcinoma	Cyclophosphamide	Mayo Clinic	NCT02650635
			Metastatic Pancreatic Adenocarcinoma			
			Recurrent Breast Carcinoma			
		2	Epithelial Ovarian Cancer	PLD	Celgene	NCT01666444
			Fallopian Tube Cancer			
			Fallopian Tube Cancer			
TLR7/8	MEDI9197	1	Solid Tumors	durvalumab	MedImmune LLC	NCT02556463
	BDC-1001	1/2	HER2 Positive Solid Tumors	Nivolumab	Bolt Biotherapeutics, Inc.	NCT04278144
	BDB001	1	Tumor, Solid	Atezolizumab	Birdie Biopharmaceuticals HK Limited	NCT04196530

### 5.1 Targeting TAMs and T cells in cancercell therapy

Immune checkpoint inhibitors have demonstrated effective anti-tumor effects by regulating T cell activity. Furthermore, their functions in regulating macrophages have also been revealed. PD-L1 is a significant immune suppressor which can regulate macrophages and T cells interaction in tumors (185). Xiong et al. reported that anti-PD-L1 therapy can not only activate CD8^+^ T cells expressing a high level of granzyme-B but also reprogram macrophages from anti-inflammatory to a pro-inflammatory phenotype, meanwhile, increasing the CD8^+^ T/Treg ratio (186). Therefore, targeting both macrophages and T cells is required for synergistic therapy.

The CD47/signal regulatory protein-α (SIRPα) cascade is an important transmembrane protein that functions as a “don’t eat me” signal, which can be delivered to macrophages (187). Depletion of SIRPα on intratumoral macrophages can enhance the therapeutic response of radiotherapy and reshape the TME from anti-inflammatory to pro-inflammatory. Furthermore, the SIRPα^-/-^ can promote high levels of pro-inflammatory factors expression, induce tumor-specific cytotoxic CD8^+^ T cells expansion and activation, and exert efficient anti-tumor immunity in colorectal and pancreatic tumors (188). Therefore, targeting CD47/SIRPα signaling may play an important role in regulating macrophages and T cells. Furthermore, recent studies have revealed that the combination of anti-PD1/PD-L1 and inhibition of CD47/SIRPα signaling developed more effective cancer immunotherapy through activating macrophages phagocytosis and antitumor effects which can further activate CD8^+^ T cells (189, 190). In addition to the CD47/SIRPα cascade, CD40 agonists re-educate TAMs into M1 macrophages to restore cancer immune surveillance (191), and the combination therapy of anti-PD1/PD-L1 and anti-CD40 also enhances anti-tumor efficacy (191, 192).

Recently, a novel nanomedicine has been constructed that can activate CD4^+^ T cells and CD8^+^ T cells and polarize the M2 macrophages to M1 macrophages, which induced potent anti-tumor immunity and has good clinical application prospects (193). Besides, Wang and colleagues found a novel cryo-thermal therapy that can induce substantial amounts of iron secretion, which promote M1 macrophage polarization through inhibiting ERK phosphorylation and the M1 macrophages can further promote CD4^+^ T cells differentiation into CD4 cytolytic T lymphocytes (CTL) (194). In addition, blockade of macrophage scavenger receptor common lymphatic endothelial and vascular endothelial receptor-1 (Clever-1) (185), ibuprofen (195), sophoridine (196) and all-trans retinoic (197) have also been demonstrated to activate endogenous antitumor CD8^+^ T cells and convert the TME from anti-inflammatory to pro-inflammatory state.

Adoptive immunotherapy with CAR-T cells has shown good clinical value on the prognosis of patients with cancer, expecially those with hematologic malignancies (198). Rodriguez and colleagues demonstrated that CAR-T cells specific for human FRβ specifically recognize and delete M2-like FRβ^+^ TAMs and enhance the anti-tumor efficiency of CAR-T cells (199). However, cytokines, including IL-6 and IL-1β released from macrophages may cause serious adverse effects of CAR-T therapy, such as cytokine release syndrome (CRS) (200, 201). CRS is thought to be the most common severe toxicity of CAR-T therapy which is characterized by high fevers, hypotension, hypoxia, sunus tachycardia and depressed cardiac function and greatly limit the broad use of CAR-T treatment (202–206). Therefore, it is urgent to find effective therapeutic strategy targeting macrophages to reduce the occurrence of CRS. Taken together, these findings suggest that novel strategies targeting both TAMs and T cells can significantly enhance anti-tumor activity.

### 5.2 Targeting TAMs and Tregs in cancer cell therapy

High-level infiltration of Tregs in TME has been demonstrated to be associated with poor prognosis (207), while the depletion of Tregs with anti-CD25 has been used in tumors and achieved preliminary results in melanoma (208), ovarian, breast and lung carcinoma (209). Currently, there is limited evidence for the use of combination therapy between macrophages and Tregs. Liver X receptor (LXR) is a member of the nuclear receptor family of transcription factors (210), studies have found that LXR agonists can obstruct tumor growth in melanoma (211), breast cancer (212), lung cancer and colon cancer (213). However, the exact mechanism of their anti-tumor activities remains undefined. Carbo and colleagues found that LXR agonist T0901317 can reduce infiltration of Tregs in tumors and TAMs expression of chemokine CCL17 which attracts Tregs migration. Furthermore, LXR agonists also inhibit IRF4 expression which further reduces the downstream genes in macrophages, such as CCL17 (214). Thus, activation of LXR might be an effective treatment in regulating the TAMs and Tregs-mediated immunosuppressive in tumors. Macrophage receptor with collagenous structure (MARCO) is a scavenger receptor expressed mainly in macrophages (215), MARCO-expressing TAMs have been demonstrated to induce Tregs proliferation and promote tumor progression in lung cancer, thus targeting MARCO with antibodies decrease Tregs frequencies and activation (216).

### 5.3 Targeting TAMs and CAFs in cancer cell therapy

Due to the highly heterogeneous of CAFs, it is difficult to target CAFs through unique markers. Thus, it is necessary to investigate the molecules and signaling pathways that affect CAFs activation and function. Studies have demonstrated that NFκB induces CAFs activation and promotes tumor epithelial-mesenchymal transition and induces chemo-resistance by expressing IL-6 and IL-8 (103, 217). Therefore, the NFκB signaling pathway may be a potential target for cancer therapy. CSF1/CSF1R signaling pathway plays a key role in regulating TAMs proliferation and polarization, many studies have confirmed the effectiveness of CSF1R inhibitors in depleting TAMs and targeting tumors (218, 219). However, Kumar et al. found that CAFs can promote polymorphonuclear myeloid-derived suppressor cells migrating into tumor tissues through secreting CXCL1 and weaken the anti-tumor effect of CSF1R inhibitors (220). Therefore, a combination of CSF1R inhibitor with blockade of macrophage recruitment may improve treatment efficacy. Furthermore, a synergistic anti-tumor effect was observed when combined anti-PD-1 with these two inhibitors.

### 5.4 Targeting TAMs and B cells in cancer therapy

The relationship between TAMs and B cells in inflammation and tumor has been reported, however, the related applications in treatment have been poorly analyzed. Affara et al. found that clearance of B cells can regulate TAMs reprogram into the M1 macrophages by using B cells-specific deletion mice, induce macrophages to express anti-tumor chemokines and activate CD8^+^ T cells in squamous carcinomas. In addition, αCD20 monoclonal antibodies, which can deplete B cells, have also been demonstrated to promote TAMs to express high levels of angiostatic and CCR chemokines, such as CXCL10, CXCL11, and CCR5, which can elevate CD8^+^ T cells infiltration and improve the response to chemotherapy. Thus, the interaction of B cells and macrophages may serve as a target for cancer cell therapy.

It is well known that CD40 agonist antibodies can reprogram M2 macrophages into M1 macrophages. Furthermore, Inoue and colleagues found that CD40-CD40L interaction can down-regulate the immunosuppressive effects of B cells on T cells and NK cells and stimulate IFN-γ production to exert anti-tumor immune response (221). Therefore, depletion of B cells and reprogramming macrophages *via* CD40 agonist antibody may have potential use in cancer treatment. Studies have confirmed that TAMs have an important role in the progression of B-cell lymphomas, such as classic Hodgkin’s lymphoma (222) and chronic lymphocytic leukemia (223). Considering the CSF1/CSF1R signaling pathway as an effective therapeutic target for depleting and reprogramming TAMs, blockade of CSF1/CSF1R signaling has been demonstrated to effectively deplete neural-like cells and control the progression of chronic lymphocytic leukemia (223). However, TAMs depletion not only induces leukemic cell death mainly through the TNF pathway, but also increases CD20^+^ leukemic cell infiltration (224). Therefore, a combination targeting TAMs and anti-CD20 mAbs may provide an effective strategy for chronic B lymphocytic leukemia. In conclusion, targeting the TAMs and B cells is also a promising therapeutic strategy for malignant tumors.

### 5.5 Targeting TAMs and neutrophils in cancer cell therapy

Dual targeting of TAMs and TANs might be an effective anti-tumor therapy strategy. The CSF-1R blockade can significantly deplete TAMs infiltration and stimulate intratumoral type I interferon signaling, which further targets the immunosuppressive TANs and elevate anti-tumor immune response during cisplatin therapy (225). In addition, voets et al. found that selective pan-allele anti-SIRPα antibody ADU-1805 has also been demonstrated to increase macrophages phagocytosis and enhance neutrophils trogocytosis, but not impact T cells activation (226). Furthermore, Ring and colleagues have revealed a new anti-human SIRPα antibody, KWAR23, which can elevate both neutrophils and macrophages’ anti-tumor activity *in vitro* and *in vivo* (227). Currently, the optimal treatment for cancers has not yet been defined. Therefore, discovering effective therapeutics targeting both macrophages and neutrophils is important for tumor patients.

IL-23 promotes M2 macrophages and neutrophils infiltration and releases immunosuppressive cytokines, such as TGF-β, IL-10 and VEGF, which reduce CD8^+^ T cells proliferation and suppress anti-tumor responses (228). Therefore, IL-23 could be a potential target for new therapeutic strategies by regulating macrophages and neutrophils simultaneously. In addition to IL-23, phospholipase D-2 (PLD2) has also been found to play a significant role in tumor progression and metastasis, and PLD was also identified to modulate macrophages and neutrophil signaling pathways (229, 230). In addition, a study found that PLD-specific inhibitors can reduce TAMs and TANs infiltration in tumors and decrease tumor growth in breast cancer, which may implicate PLD as a potential therapeutic target in the treatment of cancers (231). Besides, DKK1 was also found to inhibit TAMs and TANs infiltration in lung metastases (232).

### 5.6 Targeting TAMs and DCs in cancer cell therapy

Tumor immunotherapy with DCs vaccinations are being extensively investigated in recent years (233). The vaccines aim to enhance DCs immunogenicity and activate cytotoxic T cells (234). Some clinical trials of DCs vaccines have demonstrated that vaccines can significantly elevate the anti-tumor effectors in renal cell carcinoma (235), acute myeloid leukemia (236) and lung cancer (237). In addition, a combination of TAMs depletion and DCs vaccine has been reported to induce durable an-titumor immunity and improve survival than monotherapy in mesothelioma mouse models (238). As mentioned above, blockades of CD47/SIRPα signaling play an important role in regulating macrophages and T cells. It has also been found that anti-CD47 antibody can induce type I interferon expression in DCs and promote antigen presentation to CD8^+^ T cells (239). Though blockade of CD47/SIRPα signaling promoted macrophages phagocytosing tumor-originated mitochondrial DNA (mtDNA), it inhibited the phonological function of DCs which can reduce mtDNA degradation in DCs and activate DCs’ anti-tumor function by inducing type I interferon (240). Similarly, the CD40 agonist antibody not only reprograms M2 macrophages into M1 macrophages, but also activates DCs (147). Thus, TAMs-targeting therapy combined with DCs vaccines may be an effective strategy for regulating immune responses against tumors.

### 5.7 Targeting TAMs and NK cells in cancer cell therapy

Numerous studies have confirmed that CD47 is overexpressed in several tumor types, such as myeloma, breast cancer, leiomyosarcoma, and acute lymphocytic cancer (187, 241–243). Furthermore, CD47 is also an important marker for M2-type TAMs, and anti-CD47 therapy can reprogram TAMs to proinflammatory (M1-type) macrophages to kill tumor cells and prevent tumor metastases in human solid tumors (187). Zhang and colleagues first found differential phagocytosis effects of CD47-SIRPα inhibitors on human and mouse macrophage polarization isoforms *in vitro* (244). Although the polarization shift from the M2 to the M1 phenotype induced by anti-CD47 treatment was not verified *in vitro*, the *in vivo* results revealed that the macrophage population changed constantly and polarized towards the M1 subset with a proinflammatory immune response in the TME. Furthermore, the CD47/SIRPα signaling has also been shown to regulate NK cell functions. Overexpression of CD47/SIRPα inhibits NK cell activation and limits NK cell-mediated killing (245). Therefore, blockade of CD47/SIRPα may not only reprogram macrophages polarization, but also enhance the anti-tumor activity of NK cells.

IL-15 can potently enhance peripheral NK cells number and induce NK cells and macrophages activation (246). Furthermore, the interactions between NK cells and macrophages which can increase NK-cell activation are important for NK cells to express FcγRIV to exert cytotoxic effects under the stimulation of IL-15. Combination treatment of IL-15 and rituximab showed a better therapeutic effect which is mediated by both NK cells and macrophages to induce optimal antibody-dependent cellular cytotoxicity (246).

TAMs can be repolarized into the M1 phenotype *via* activating Toll-like receptor (TLR) and stimulate NK cell activity through expressing immunostimulatory cytokines IL-12 (247). Combination therapy used anti-tumor antibody, IL-12 and anti-PD-1 can induce macrophages repolarized into a M1 phenotype and promote NK cell proliferation, activation and cytotoxicity (248). Reprogrammed macrophages and NK cells trigger lymphocytes’ recruitment into tumors *via* secreting IFN-γ and facilitate tumor vascular normalization which greatly improved the anti-tumor efficacy. Thus, therapies targeting innate cell activation, such as macrophages and NK cells, may initiate T cell-mediated anti-tumor immune responses.

## 6 Perspectives and conclusions

Recently, increasing studies have revealed the role of TME in tumorigenesis, progression, and response to treatment (194, 249). Besides, substantial single-cell-related studies have also revealed that TAMs are one cell subgroup of the most abundant components in TME with important functions (7, 250). According to the current understanding, there are mainly two sources of TAMs, including tumor-resident macrophages and bone marrow-derived macrophages which are regularly further polarized into M1 and M2 phenotypes. Tumor-resident macrophages and M2-type TAMs normally play a pro-tumor role, while M1-type TAMs inhibit tumor development (40). Notably, interactions between TAMs and other immune cells in TME significantly influence tumor progression. Many current strategies for cancer treatment influence TME and the TME changes are associated with the therapeutic efficacy. Thus, novel strategies targeting TAMs and other immune cells and their crosstalk will be a promising approach to block or eradicate the tumor.

Currently, CAR-T cell therapies still occupy the major position, but cell therapy modalities that rely on other immune cells have solidified their growth in the past year (179). Given that the important role of TAMs in cancer progression and response to anti-cancer treatment, TAMs-based cell therapy may also be a promising direction. Furthermore, recent studies have revealed that regulating TAMs can significantly inhibit cancer progression and enhance the therapeutic effects of other treatments (186). Recently, CAR-M therapy and targeting strategies regulating TAMs or crosstalk between TAMs and other immune cells have been well studied and achieved encouraging results in cancer treatment.

Conclusively, the effects of TAMs on the initiation and progression of various cancers can be realized in multiple approaches. As the most abundant component in TME, TAMs had strong associations with other immune cells and these interactions exert important effects on cancer progression. Furthermore, targeting TAMs and the interactions with other immune cells can exert antitumor effects. Therefore, TAMs-related immunotherapy is a promising approach to improve therapeutic efficacy for cancer treatment.

## Author contributions

CL provided the detection of the manuscript. ML and PJ wrote and edited the manuscript. ML and SW collected associated data. CL and ML drew the figures and tables. CL and JW guided the preparation of this manuscript. All authors read and approved the final manuscript. All authors contributed to the article and approved the submitted version.

## Glossary

**Table:**

TAMs	Tumor-associated macrophages
TME	tumor microenvironment
EMT	epithelial–mesenchymal transition
TNF-α	tumor necrosis factor-α
Arg-1	arginase 1
A2R	A2 adenosine receptor
VEGF	vascular endothelial growth factor
NO	nitric oxide
CCL-17	Chemokine (C-C motif) ligand 17
NF-κB	nuclear factor-κB
APCs	antigen-presenting cells
DCs	dendritic cells
TLR	toll-like receptor
LPS	lipopolysaccharide
IFN-γ	interferon-γ
IL-12	interleukin-12
ROS	reactive oxygen species
Th2	T-helper
TGF-β	transforming growth factor-β
EGF	epidermal growth factor
Fgf2	fibroblast growth factor
Igf1	insulin-like growth factor-1
RAPA	rapamycin
STING	Stimulator of interferon genes
DHHL	Hispanolone derivative 8, 9-dehydrohispanolone-15, 16-lactol
SOCS	Suppressors of cytokine signaling
CARKL	Carbohydrate kinase-like protein
HIF1α	Hypoxia Inducible Factor 1α
PDK1	pyruvate dehydrogenate kinase 1
PKM1	Pyruvate kinase M1
PHD2	prolyl hydroxylase domain 2
HCC	hepatocellular carcinoma
MMPs	matrix metalloproteinases
CHI3L1	chitinase 3-like protein 1
MAPK	mitogen-activated protein kinase
3D	three-dimensional
CCR2	CCL2/CC chemokine receptor 2
TF	tissue factor
COX-2	cyclooxygenase-2
NK	natural killer
KIR	immunoglobulin-like receptor
Tregs	regulatory T cells
MPE	malignant pleural effusion
CCL2	CC-chemokine ligand 2
PDA	pancreatic ductal adenocarcinoma
CTLs	CD8+ cytotoxic T lymphocytes
SIRPα	signal regulatory protein-α
CpG ODN	Oligodeoxynucleotides containing CpG motifs
TLR9	Toll-like receptor 9
TLRs	Toll like receptors
